# MicroRNA-99a induces G1-phase cell cycle arrest and suppresses tumorigenicity in renal cell carcinoma

**DOI:** 10.1186/1471-2407-12-546

**Published:** 2012-11-23

**Authors:** Li Cui, Hua Zhou, Hu Zhao, Yaojun Zhou, Renfang Xu, Xianlin Xu, Lu Zheng, Zhong Xue, Wei Xia, Bo Zhang, Tao Ding, Yunjie Cao, Zinong Tian, Qianqian Shi, Xiaozhou He

**Affiliations:** 1 Department of Urology, The Third Affiliated Hospital of Soochow University, 185 Juqian Street, Changzhou, 213003, China; 2 Department of Nephrology, The Third Affiliated Hospital of Soochow University, Changzhou, China; 3 Department of Urology, The Affiliated Jiangyin Hospital of Southeast University Medical College, Wuxi, China; 4 Comprehensive Laboratory, The Third Affiliated Hospital of Soochow University, Changzhou, China

**Keywords:** MicroRNA-99a, mTOR, Renal cell carcinoma

## Abstract

**Background:**

A growing body of evidence suggests that microRNAs (miRNAs) play an important role in cancer diagnosis and therapy. MicroRNA-99a (miR-99a), a potential tumor suppressor, is downregulated in several human malignancies. The expression and function of miR-99a, however, have not been investigated in human renal cell carcinoma (RCC) so far. We therefore examined the expression of miR-99a in RCC cell lines and tissues, and assessed the impact of miR-99a on the tumorigenesis of RCC.

**Methods:**

MiR-99a levels in 40 pairs of RCC and matched adjacent non-tumor tissues were assessed by real-time quantitative Reverse Transcription PCR (qRT-PCR). The RCC cell lines 786-O and OS-RC-2 were transfected with miR-99a mimics to restore the expression of miR-99a. The effects of miR-99a were then assessed by cell proliferation, cell cycle, transwell, and colony formation assay. A murine xenograft model of RCC was used to confirm the effect of miR-99a on tumorigenicity in vivo. Potential target genes were identified by western blotting and luciferase reporter assay.

**Results:**

We found that miR-99a was remarkably downregulated in RCC and low expression level of miR-99a was correlated with poor survival of RCC patients. Restoration of miR-99a dramatically suppressed RCC cells growth, clonability, migration and invasion as well as induced G1-phase cell cycle arrest in vitro. Moreover, intratumoral delivery of miR-99a could inhibit tumor growth in murine xenograft models of human RCC. In addition, we also fond that mammalian target of rapamycin (mTOR) was a direct target of miR-99a in RCC cells. Furthermore, siRNA-mediated knockdown of mTOR partially phenocopied the effect of miR-99a overexpression, suggesting that the tumor suppressive role of miR-99a may be mediated primarily through mTOR regulation.

**Conclusions:**

Collectively, these results demonstrate for the first time, to our knowledge, that deregulation of miR-99a is involved in the etiology of RCC partially via direct targeting mTOR pathway, which suggests that miR-99a may offer an attractive new target for diagnostic and therapeutic intervention in RCC.

## Background

Renal cell carcinoma (RCC) is the most common neoplasma of the kidney in adults accounting for about 3% of adult malignancies [1], with having the highest mortality rate at over 40% [2]. The 5-year survival of RCC is estimated to be approximately 55% [3], and that of metastatic RCC is approximately 10% [4]. Surgical resection is still the only definitive treatment for RCC, but after the curative nephrectomy, 20–40% patients will develop recurrence [5]. This is mainly a consequence of the fact that RCC is resistant to both chemotherapy and radiotherapy [6]. So no adjuvant therapy is available in clinical routine. Moreover, the absence of biomarkers for early detection and follow-up of the disease complicate the on-time diagnosis. Therefore, novel tumor markers that have higher sensitivity and reliability and effective therapeutic methods are urgently needed for RCC.

MicroRNAs (miRNAs) are a class of naturally occurring, non-coding, short single stranded RNAs, in the size range 19–25 nucleotides, that regulate gene expression at the post-transcriptional level, by binding through partial sequence homology, to the 3^′^untranslated region (3′UTR) of mammalian target mRNAs and causing translational inhibition and/or mRNA degradation [7]. It has been firmly established that miRNAs control various key cellular processes, such as proliferation, cell cycle, differentiation, and tumorigenesis [8]. In recent years, numerous studies have shown aberrant expression of miRNAs in human cancers [9], including RCC [10], some of which function as tumor suppressor genes or oncogenes [11]. Due to their tissue- and disease-specific expression patterns and tremendous regulatory potential, miRNAs are being identified as diagnostic and prognostic cancer biomarkers, as well as additional therapeutic tools [12].

It has been reported that miR-99a is transcribed from the commonly deleted region at 21q21 in human lung cancers [13], and that miR-99a is downregulated in ovarian carcinoma [14], squamous cell carcinoma of the tongue [15], squamous cell lung carcinoma [16], hepatocellular carcinoma [17], bladder cancer [18], prostate cancer [19] and childhood adrenocortical tumors [20]. These findings indicate that miR-99a is widely downregulated in human cancers, suggesting a potential role of miR-99a as a tumor suppressor. However, up to date, there are no studies of miR-99a in RCC. Thus, we concentrated on miR-99a in RCC.

The present study was undertaken to examine the expression of miR-99a in RCC cell lines and tissues, assess the impact of miR-99a on RCC cells and RCC xenograft modle, and identify target genes for miR-99a that might mediate their biological effects. In this study, we observed that miR-99a was remarkably downregulated in RCC cell lines and tissues and correlated with overall survival of RCC patients. Restoration of miR-99a induced G1-phase cell cycle arrest in vitro and dramatically suppressed tumorigenicity of RCC in vitro and in vivo. In addition, with the help of a bioinformatic analysis, we found that the mammalian target of rapamicin (mTOR), a key promoter of cell growth, was a direct target of miR-99a in RCC cells. Furthermore, siRNA-mediated knockdown of mTOR partially phenocopied miR-99a restoration suggesting that the tumor suppressive role of miR-99a may be mediated primarily through mTOR regulation. Our study suggests that miR-99a may offer an attractive new target for diagnostic and therapeutic intervention in RCC.

## Methods

### Tissue samples

The study was approved by the ethics committee of the Third Affiliated Hospital of Soochow University. Written informed consent was obtained from each patient for the use of material to research purposes. All tissue samples (40 pairs) contained more than 80% tumor cells were obtained from the Department of Urology, the Third Affiliated Hospital of Soochow University, China. Tumor tissues were harvested during partial or radical nephrectomy and confirmed renal cell carcinoma by pathological study post operatively. Adjacent non-tumor tissues were also resected simultaneously, and half of them were sent for pathological inspection to rule out contamination of tumor. Tissue samples were immediately frozen in liquid nitrogen until analysis.

### Cell lines and cell culture

The nonmalignant SV-40 immortalized renal cell line HK-2 was obtained from KeyGen Biotech (Nanjing, China), which was maintained in DMEM with 10% FBS. The human renal cancer cell lines 786–0 and OS-RC-2 were obtained from the Chinese Academy of Sciences Cell Bank, which were maintained in RPMI 1640 with 10% FBS. All cell lines were cultured at 37°C in a humidified incubator (5% CO_2_).

### miRNA/siRNA transfections

2^′^-O-methyl (2^′^-O-Me) oligonucleotides were chemically synthesized by GenePharma Biotechnology (Shanghai, China). The sequences were as follows: miR-99amimics: (forward) 5^′^-AACCCGUAGAUCCGAUCUUGUG-3^′^,(reverse) 5^′^-CAAGAUCGGAUCUACGGGUUUU-3^′^; mTOR-siRNA: (forward) 5^′^-ACCAUGAACCAUGUCCUAAGCUGTG-3^′^, (reverse) 5^′^-CACAGCUUAGGACAUGGUUCAUGGUAU-3^′^; negative control (NC) : (forward) 5^′^-UUCUCCGAACGUGUCACGUTT-3^′^, (reverse) 5^′^-ACGUGACACGUUCGGAGAATT-3^′^. Cells at 70%–80% confluence were transfected with miR-99a mimics, mTOR-siRNA or negative control (NC) using Lipofectamine 2000 (Invitrogen) according to the manufacturer’s protocol.

### RNA isolation and real-time qRT- PCR

Total RNAs were isolated from RCC tissues and cell lines using TRIzol reagent (Invitrogen, USA) for miRNA analyses. MiR-99a real-time qRT-PCR was performed by the TaqMan miRNA assays (Applied Biosystems, USA) and U6 was used as an internal control. PCR cycles were as follows: initial denaturation at 95°C for 10 minutes, followed by 40 cycles at 95°C for 15 seconds and 60°C for 1 minute. The relative miRNA expression was calculated using the 2^-△△Ct^ method.

### Cell proliferation assay

786–0 and OS-RC-2 cells were transfected with the miR-99a mimics, mTOR-siRNA or negative control (NC) for 48 hours and then seeded at 2000 cells per well in 96-well plates. 10 μl CCK-8 solution was added to 100 μl culture media per well above and then the plate was incubated for at 37°C 1.5 hours. The absorbance was measured at 450 nm using a Vmax microplate spectrophotometer (Molecular Devices, Sunnyvale, CA). Each sample was assayed in triplicate. This procedure was repeated at 24, 48, 72 and 96 hours after transfection.

### Colony formation assay

786-O and OS-RC-2 cells were transfected with the miR-99a mimics, mTOR-siRNA or negative control (NC) for 24 hours and then seeded for colony formation in 6-well plates at 200 cells per well. After 15 days, cells were stained with Giemsa, and then colonies were counted only if a single clone contained more than 100 cells. Each assay was performed in triplicate.

### Cell cycle assay

Transfected RCC cells in the log phase of growth were collected and fixed in 75% ethanol at −20°C for 16 hours. For cell cycle analysis, transfected cells were stained with propidium iodide and examined with a fluorescence-activated cell sorting (FACS) flow cytometer (BD Biosciences, San Jose,CA), and DNA histograms were analyzed with modified software. Each test was repeated in triplicate.

### Cell migration and invasion

786-O and OS-RC-2 cells were transfected with the miR-99a mimics, mTOR-siRNA or negative control (NC), cultivated for 48 hours, and transferred on the top of Non-matrigel-coated/ Matrigel-coated chambers (24-well insert, 8-μm pore size, BD Biosciences, San Jose, USA) in a serum-free RPMI 1640 and the medium containing 30% fetal calf serum was added to the lower chamber as a chemoattractant. After incubation for 48 hours, non-migrated/non-invaded cells were removed from the upper well with cotton swabs while the migrated/invaded cells were then fixed with 4% paraformaldehyde, stained with 0.1% crystal violet, and photographed (×200) in five independent fields for each well. Each test was repeated in triplicate.

### Nude mouse tumor xenograft model

All experimental procedures involving the use of animals were in accordance with the Guide for the Care and Use of Laboratory Animals and were approved by the ethics committee of the Third Affiliated Hospital of Soochow University. Nude mice (5- to 6-week old; SLAC ANIMAL, China; n = 12) received subcutaneous injections of 3 × 10^6^ 786-0 cells in the right flank area in a volume of 200 μl. Once palpable tumors developed, the volume of tumor was measured with a caliper every 4 days, using the formula: volume = (length × width^2^)/2. When the tumor volume reached an average volume of 75 to 100 mm^3^, the mice were randomly divided into two groups (six mice per group). These mice were then treated with 200 pmol miR-99a or NC mimics in 10 μl Lipofectamine 2000 through a local injection of the xenograft tumor at multiple sites.

### Luciferase activity assay

The portion of the 3^′^UTR region (908 bp) of human mTOR gene containing the miR-99a binding site was amplified by PCR using the following primers: mTOR-3^′^UTR-F: 5^′^-CTTTCAGAAACTGGAGGCCCAG-3^′^ and mTOR-3^′^UTR-R: 5^′^-TGGTGTCTAGACATGGCTACACTTTATAC-3^′^. This portion was amplified and cloned into the XbaI site of the pGL3-control vector (Promega, USA), downstream of the luciferase gene, to generate the plasmids pGL3-WT-mTOR-3^′^UTR. pGL3-MUT-mTOR- 3^′^UTR was generated from pGL3-WT-mTOR-3^′^UTR by deleting the binding site for miR-99a “UACGGGU”. For the luciferase reporter assay, the 786–0 and OS-RC-2 cell lines were co-transfected with luciferase reporter vectors and miR-99a mimics using Lipofectamine 2000. A 1-ng pRL-TK Renilla Luciferase construct was used for normalization. After 48 hours, luciferase activity was analyzed by the Dual-Luciferase Reporter Assay System according to the manufacturer’s protocols (Promega, Madison, USA).

### Western blotting analysis

Total protein was collected by Total Protein Extraction Kit (KeyGen, China); 30 μg of protein per lane was separated by 12% SDS-polyacrylamide gel and transferred to PVDF membrane. The membrane was blocked in 5% skim milk for 2 hours and then incubated with a specific antibody for 2 hours. The antibodies used in this study were: primary antibodies against Cyclin-D1, Cyclin-D2, Cyclin-E (Bioworld,Nanjing, China), mTOR, phospho-mTOR, p70S6K, phospho-p70S6K, 4E-BP1 and phospho-4E-BP1 (Cell Signaling Technology, USA). GAPDH and β-actin (Bioworld, Nanjing, China) on the same membrane was used as a loading control. The specific protein was detected by a BCA Protein Assay Kit (KeyGen, China). The band density of specific proteins was quantified after normalization with the density of GAPDH or β-actin.

### Statistical analysis

Data are presented as the mean ± standard deviation (SD) from at least three independent experiments. Student’s *t* test and one-way analysis of variance (ANOVA) were used to analyze significant differences using SPSS 17.0 (SPSS Inc., USA). All *P* < 0.05 were marked with *, and *P* < 0.01 with **.

## Results

### miR-99a is downregulated and correlates with overall survival in renal cell carcinoma

**Figure 1:**
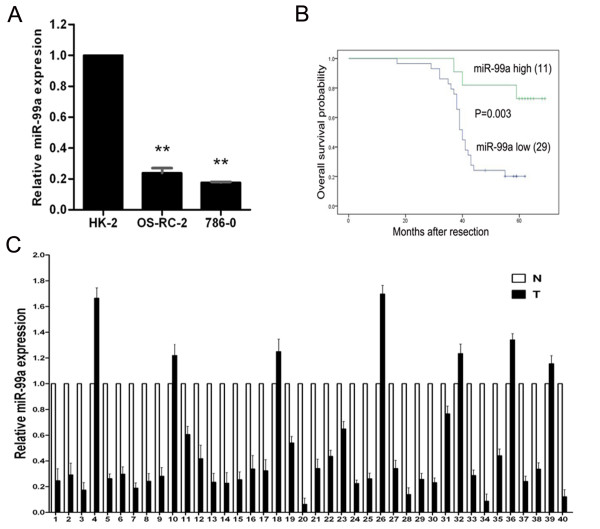
**MiR-99a is downregulated in renal cell carcinoma.** (**A**) Real-time qRT-PCR analysis of relative miR-99a expression levels in RCC cell lines (786-O and OS-RC-2) and normal immortalized renal cell line (HK-2). (**B**) Relative miR-99a expression levels in 40 pairs of RCC and their matched adjacent non-tumor tissues as assessed by real-time qRT-PCR. MiR-99a was considered to be significantly downregulated only if the calculated fold-change was less than 0.5 in the tumor tissue compared with the matched adjacent non-tumor tissue. (**C**) Correlation of miR-99a expression with overall survival in RCC patients. Overall survival of RCC patients were analyzed by Kaplan-Meier analysis in SPSS 17.0. Relative miR-99a level was assessed by real-time qRT-PCR and T/N = 0.5 was chosen as the cut-off point for separating miR-99a high-expression tumors (n = 11; T/N > 0.5) from miR-99a low-expression cases (n = 29; T/N < 0.5). Data were normalized to U6 control and are represented as mean ± standard deviation (SD) from three independent experiments. T means RCC tissues. N means matched adjacent non-tumor tissues. **, *P* < 0.01.

To identify the expression of miR-99a in RCC, we firstly performed real-time qRT-PCR using the renal cell line HK-2 and RCC cell lines 786–0 and OS-RC-2 and found that miR-99a expression in RCC cell lines (786–0 and OS-RC-2) was significantly lower than that in HK-2 (Figure 1A). Then we analysed miR-99a expression in clinical samples. Patient and tumor characteristics are showed in Table 1. Total RNA was extracted from 40 pairs of RCC and their adjacent non-tumor tissues and real-time qRT-PCR was performed. MiR-99a was considered to be significantly downregulated only if the calculated fold-change was less than 0.5 in the tumor tissue compared with the matched adjacent non-tumor tissue. As consistent with the results in cell lines, the expression of miR-99a was remarkably downregulated in RCC tissues (29/40, 72.5%), compared with matched adjacent non-tumor tissues (Figure 1B). Notably, dramatic downregulation of miR-99a was observed in 50% (9/18) cases of low stage (pT1 + pT2) and 91% (20/22) cases of high stage (pT3 + pT4) RCC. These results indicate that miR-99a expression possibly correlates with pathologic stage of RCC. To investigate whether downregulation of miR-99a in RCC tissues correlated with overall survival of RCC patients, we performed statistical analysis with Kaplan-Meier method. As shown in Figure 1C, lower miR-99a expression level in RCC tissues dramatically correlated with decreased overall survival of RCC patients. These data suggest that miR-99a may be a predictor for prognosis of RCC patients.

**Table 1:** Patients and tumor characteristics (n = 40; 2005–2007)

**No.**	**Age**	**Sex**	**Pathologic Diagnosis**	**pT Stage**	**Grade**
1	62	female	clear cell	T1b	2
2	61	male	clear cell	T3b	2
3	64	male	clear cell	T4	3
4	69	female	clear cell	T1a	1
5	73	male	clear cell	T2b	4
6	62	female	clear cell	T3a	3
7	53	male	clear cell	T2b	2
8	57	male	clear cell	T3b	3
9	42	male	clear cell	T2b	3
10	64	male	clear cell	T2a	2
11	70	female	clear cell	T1b	2
12	68	female	clear cell	T3a	3
13	48	male	clear cell	T3a	3
14	78	male	clear cell	T1b	2
15	65	male	clear cell	T3a	3
16	38	female	clear cell	T3a	2
17	56	male	clear cell	T3b	3
18	44	female	clear cell	T3a	2
19	63	female	clear cell	T2a	1
20	69	male	clear cell	T4	2
21	55	male	clear cell	T3b	3
22	60	male	clear cell	T3a	2
23	38	female	clear cell	T1a	1
24	45	male	clear cell	T3b	2
25	62	female	clear cell	T3b	2
26	41	male	clear cell	T1b	3
27	60	female	clear cell	T2b	3
28	63	male	clear cell	T3b	2
29	35	male	clear cell	T1b	4
30	77	male	clear cell	T3a	3
31	64	male	clear cell	T2a	3
32	42	female	clear cell	T1a	1
33	68	female	clear cell	T3b	3
34	71	male	clear cell	T4	4
35	52	male	clear cell	T2b	2
36	62	female	clear cell	T3b	2
37	69	male	clear cell	T3a	3
38	73	male	clear cell	T3b	3
39	50	female	clear cell	T2a	2
40	38	male	clear cell	T1a	3

### miR-99a suppresses tumorigenicity in vitro

**Figure 2:**
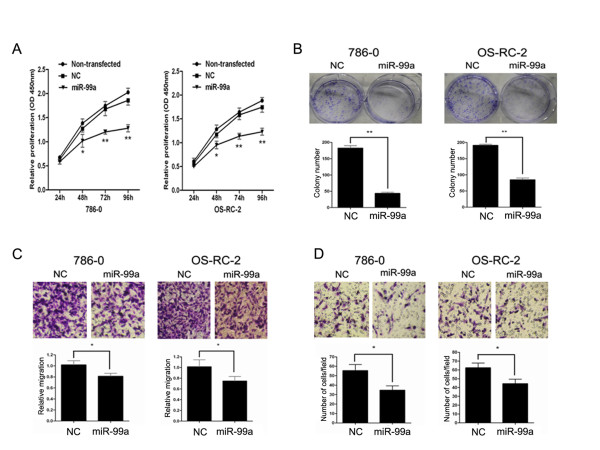
**MiR-99a suppresses tumorigenicity in vitro.** 786-O and OS-RC-2 cells were transfected with miR-99a or NC followed by functional assays. (**A**) Cell proliferation analysis of transfected RCC cells and non-transfected RCC cells by CCK-8 assay at 24 , 48 , 72 and 96 hours after transfection. (**B**) Colony formation assay of transfected RCC cells at 15 days after transfection. (**C**, **D**) Migration and invasion analysis of transfected RCC cells by transwell assay at 48 hours after transfection. Data are represented as mean ± SD from three independent experiments. *, *P* < 0.05. **, *P* < 0.01.

The reduced expression of miR-99a in RCC prompted us to identify whether miR-99a functions as a tumor suppressor. To investigate the function of miR-99a, we restored miR-99a in RCC cell lines. 786–0 and OS-RC-2 cells were transfected with miR-99a or NC, and then functional assays were performed. CCK-8 assay showed that mir-99a restoration was more potent than their NC transfectants in inhibiting the proliferation of RCC cells. (Figure 2A). As shown in Figure 2B, compared with NC transfectants, miR-99a-restored RCC cells displayed notably fewer and smaller colonies. Transwell migration and invasion assays showed that the migration (Figure 2C) and invasion (Figure 2D) of miR-99a-restored RCC cells were reduced compared with their NC transfectants, respectively. These observations suggest that miR-99a restoration suppresses the tumorigenicity of RCC cells in vitro.

### miR-99a induces G1-phase cell cycle arrest

**Figure 3:**
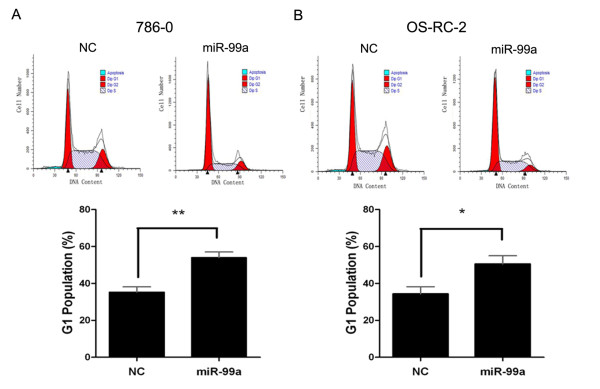
**MiR-99a induces G1-phase cell cycle arrest.** (**A**) Cell cycle analysis of transfected 786-O cells by FACS. Cells which were transfected with miR-99a showed an increased G1-phase population compared with NC transfectants. (**B**) Cell cycle analysis of transfected OS-RC-2 cells by FACS. Cells which were transfected with miR-99a showed an increased G1-phase population compared with NC transfectants. Data are represented as mean ± SD from three independent experiments. *, *P* < 0.05. **, *P* < 0.01.

To investigate the role of miR-99a in cell cycle progression, we restored miR-99a in RCC cells. 786-O and OS-RC-2 cells were transfected with miR-99a or NC. Cell cycle assay showed that mir-99a-restored RCC cells had a significant increase in G1-phase population as compared with NC transfectants (Figure 3A, B). Additionally, we also examined the effect of miR-99a on apoptosis and found that miR-99a restoration could hardly influence apoptosis in RCC cell lines (data not shown). These findings indicate that miR-99a induces G1-phase cell cycle arrest in RCC cell lines.

### miR-99a suppresses tumor growth in vivo

**Figure 4:**
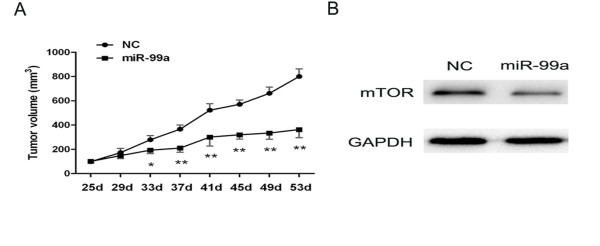
**MiR-99a suppresses tumor growth in vivo.** (**A**) 786-O cells were subcutaneously injected into nude mice to form solid, palapable tumors (day 25), following which synthetic miR-99a or NC mimics were intratumorally delivered for 4 weeks. Tumor volumes following miR-99a administration were significantly reduced compared with the control mice. (**B**) After tumor xenografts were intratumorally delivered synthetic miR-99a or NC mimics for 4 weeks, we extraced the protein and performed Western blot. We found that intratumoral delivery of synthetic miR-99a induced a makedly inhibition of mTOR expression compared with control mice. Data are represented as mean ± SD. *, *P* < 0.05. **, *P* < 0.01.

Because the in vitro data demonstrated that miR-99a harbored antitumorigenic properties in RCC, we conducted a proof-of-principle experiment, in which a 786–0 xenograft model was used to confirm the effect of miR-99a on tumorigenicity in vivo. As shown in Figure 4A, twenty-five days following 786–0 cells subcutaneous inoculation, the mean tumor volume of the mice in the control and treated groups was 98 and 100 mm^3^, respectively. Then, miR-99a or NC mimics was repeatedly administered by intratumoral injections every 3 days for 4 weeks. At the end of the experiment, intratumoral delivery of synthetic miR-99a induced a specific inhibitory response and robustly interfered with tumor growth compared with control mice. In addition, We detected the expression of mTOR in tumor xenografts after miR-99a injection by Western blot, we found that intratumoral delivery of synthetic miR-99a makedly suppressed mTOR expression compared with control mice (Figure 4B). These results suggest that restoration of miR-99a suppresses tumor growth in vivo and could serve as a therapeutic tool in RCC therapy.

### mTOR is a target of miR-99a

**Figure 5:**
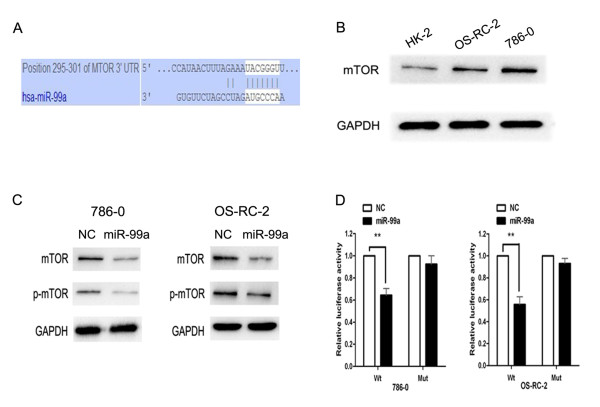
**MTOR is a target of miR-99a.** (**A**) Sites of miR-99a seed matches in the mTOR 3^′^ UTR. (**B**) Expression of mTOR protein were detected by western blotting assay in RCC cell lines (786-O and OS-RC-2) and normal immortalized renal cell line (HK-2). (**C**) Expression of mTOR and p-mTOR protein were detected by western blotting assay in RCC cell lines (786-O and OS-RC-2) after 48 hours of transfection with miR-99a or NC. (**D**) Luciferase constructs were transfected into 786-O and OS-RC-2 cells transduced with miR-99a. Luciferase activity was determined 48 hours after transfection. The ratio of normalized sensor to control luciferase activity is shown. Data are represented as mean ± SD from three independent experiments. **, *P* < 0.01.

To explore the mechanisms by which miR-99a regulates the tumorigenicity of RCC, we performed a bioinformatic search (Targetscan, Pictar and MICROCOSM) for putative targets of miR-99a and found 3^′^UTR of mTOR containing the highly conserved putative miR-99a binding sites (Figure 5A). As mentioned above, miR-99a was remarkably downregulated in RCC cell lines (Figure 1A). Western blotting analysis found a clear upregulation of mTOR protein in RCC cell lines compared with HK-2 (Figure 5B). So, there was an inverse correlation between miR-99a levels and mTOR protein. To show that miR-99a participated in the regulation of mTOR expression, we restored miR-99a in RCC cells. 786–0 and OS-RC-2 cells were transfected with miR-99a or NC. The enforced expression of miR-99a in RCC cell lines led to a decrease in mTOR protein and also led to a decrease in phospho-mTOR (p-mTOR) protein, compared with NC transfectants (Figure 5C). To ascertain the direct miR-99a-mTOR interaction, we created pGL3-WT-mTOR-3^′^UTR and pGL3-MUT- mTOR-3^′^UTR plasmids. Dual-luciferase reporter assay revealed that restoration of miR-99a led to a marked decrease in luciferase activity of pGL3-WT-mTOR-3^′^UTR plasmid in 786–0 and OS-RC-2 cells but did not change luciferase activity of pGL3-MUT- mTOR-3^′^UTR (Figure 5D). Taken together, these findings showed a direct interaction between miR-99a and mTOR mRNA in RCC cell lines.

### mTOR pathway is involved in miR-99a mediated G1/S transition

**Figure 6:**
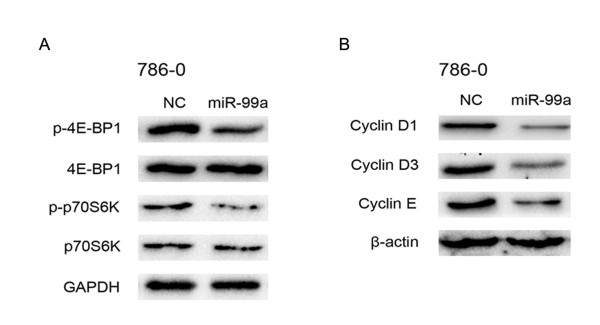
**MTOR pathway is involved in miR-99a mediated G1/S Transition.** (**A**) Expression of p70S6K, p-p70S6K, 4E-BP1 and p-4E-BP1 were detected by western blotting assay in 786-O cells after 48 hours of transfection with miR-99a or NC. (**B**) Expression of cyclin D1, cyclin D3 and cyclin E were detected by Western blotting assay in 786-O cells after 48 hours of transfection with miR-99a or NC.

To evaluate whether mTOR pathway is implicated in miR-99a induced G1-phase arrest, downstream substrates of mTOR pathway were investigated after restoration of miR-99a in 786–0 cells. We detected ribosomal protein S6 kinase, 70 kDa (P70S6K), phospho-p70S6K (p-p70S6K), Eukaryotic translation initiation factor 4E-binding protein 1 (4E-BP1) and phospho-4E-BP1 (p-4E-BP1) expression by western blotting analysis. As shown in Figure 6A, compared with NC transfectants, the expression of p-p70S6K and p-4E-BP1 were downregulated in miR-99a-restored 786–0 cells, which suppressed the activation of sequential signaling cascades involved in synthesis of several G1/S transition-related molecules [21,22]. Then we detected the expression of cyclin D1, cyclin D3 and cyclin E in miR-99a-restored 786–0 cells. Western blotting analysis showed that cyclin D1, cyclin D3 and cyclin E expression were also downregulated (Figure 6B), which may be attributed to attenuated p-P70S6K and p-4E-BP1. These results demonstrate that mTOR pathway is involved in miR-99a mediated G1/S Transition. 

### mTOR knockdown partially phenocopies miR-99a restoration in renal cell carcinoma cells

**Figure 7:**
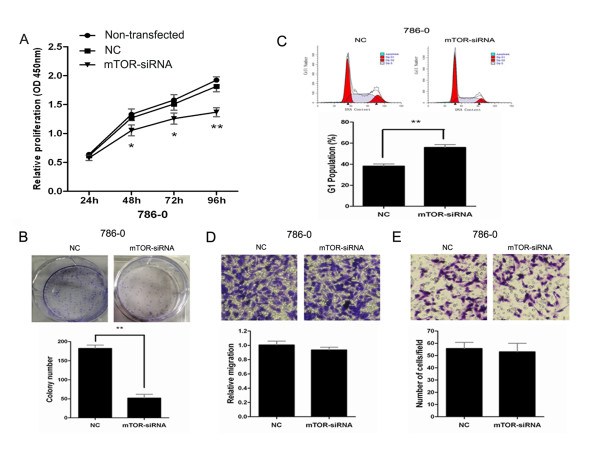
**MTOR knockdown partially phenocopies miR-99a restoration in renal cell carcinoma cells.** 786-O cells were transfected with mTOR-siRNA or NC followed by functional assays. Cell proliferation assay by CCK-8 (**A**), colony formation assay (**B**), cell cycle analysis by FACS (**C**), transwell-migration assay (**D**) and transwell-invation assay (**E**) in 786-O cells transfected with mTOR- siRNA or NC. We also detected the proliferation of non-transfected 786-O cells. Data are represented as mean ± SD from three independent experiments. *, *P* < 0.05. **, *P* < 0.01.

To further reveal mechanisms underlying this tumor suppressive effect of miR-99a, we knockdowned mTOR in RCC cells. 786–0 cells were transfected with mTOR-siRNA or NC, and then functional assays were performed. As expected, compared with NC transfectants, mTOR-knockdowned 786–0 cells showed a decrease in the proliferation and colony formation and an increase in the G1-phase population (Figure 7A–C), similar to the phenotype observed upon miR-99a restoration in 786–0 cells. However, the migration and invasion of mTOR- knockdowned 786–0 cells were not decreased compared with NC transfectants (Figure 7D, E), which suggests that the regulation of miR-99a on migration and invasion in RCC cells is not likely related to mTOR inhibition. Taken together, we conclude that the tumor suppressive role of miR-99a may be mediated partially through mTOR pathway regulation.

## Discussion

Previous studies have reported that miR-99a participated in tumorigenesis of several tumor type,including hepatocellular carcinoma [17], prostate cancer [19], childhood adrenocortical tumors [20] and lung cancer [23]. However, in this study, we demonstrate for the first time that miR-99a is implicated in the carcinogenesis of RCC. Compared with nonmalignant immortalized renal cell line HK-2, the expression of miR-99a was significantly downregulated in RCC cell lines 786–0 and OS-RC-2. As consistent with the results in cell lines, detection of miR-99a in RCC tissues also pointed to a dramatic attenuation of miR-99a expression in 72.5% (29/40) of RCC tissues. Notably, dramatic downregulation of miR-99a was observed in 50% (9/18) cases of low stage (pT1 + pT2) and 91% (20/22) cases of high stage (pT3 + pT4) RCC. In addition, lower miR-99a expression level in RCC tissues significantly correlated with reduced overall survival in RCC patients. These results indicate that miR-99a may serve as a potential predictor for prognosis of RCC patients. A limitation to our study was the relatively small number of clinical samples at our disposal. Further studies with more clinical samples are warranted.

The reduced expression of miR-99a in RCC prompted us to identify whether miR-99a functions as a tumor suppressor. We found that restoration of miR-99a suppressed cell growth, clonability, migration and invasion and induced G1-phase cell cycle arrest in vitro. Moreover, intratumoral delivery of miR-99a was sufficient to trigger in vivo regression of tumor growth in RCC xenograft model. These findings suggest that miR-99a plays a tumor suppressive role and may be a therapeutic intervention in RCC. It has been reported that overexpression of miR-99a inhibits the growth of prostate cancer cells and decreases the expression of prostate-specific antigen (PSA) [19]. In addition, restoration of miR-99a dramatically suppresses tumor cell growth in lung cancer [23]. Recently, Li *et al.* reported that restoration of miR-99a significantly inhibits hepatocellular carcinoma cell growth in vitro by inducing the G1 phase cell cycle arrest [17]. All these reports support our findings in RCC. However, Li *et al.* also reported that restoration of miR-99a could hardly influence the metastasis of hepatocellular carcinoma cell lines [17], inconsistent with our findings in RCC. Although the actual reasons are currently unclear, this inconsistency might be due to the different tumor type and cellular context.

With the help of bioinformatics prediction and sequential experimental demonstration, mTOR was identified as a direct target of miR-99a in RCC. MTOR signaling pathway is a key signal-transduction system that links multiple receptors and oncogenic molecules to diverse cellular functions and is inappropriately activated in many human cancers [24,25]. MTOR signaling pathway plays a crucial role in the regulation of cell growth, protein translation, metabolism, cell invasion, and cell cycle [26]. Major downstream targets of mTOR are p70S6K and 4E-BP1, which is activated by mTOR and then dissociates from the eukaryotic translation factor (eIF-4E) and activates protein synthesis [27]. Overexpression or overactivation of mTOR may strengthen the signals passed down by mTOR signaling pathway, which will cause over-phosphorylation of the downstream molecules p70S6K and 4E-BP1. Once phosphorylated, p70S6K and 4E-BP1 can promote protein synthesis [17]. Thus, several cell-cycle related proteins including cyclin D1, cyclin D3 and cyclin E [21,22], will be excessively upregulated which resulted in the progression of cell cycle. We restored miR-99a in 786–0 cells and found that the expression of p-p70S6K, p-4E-BP1, cyclin D1, cyclin D3 and cyclin E are really downregulated, consistent with the previous reports in hepatocellular carcinoma [17]. Therefore, activation of the mTOR pathway provides tumor cells with a growth advantage by promoting protein synthesis [28].

To further elucidate mechanisms underlying the tumor suppressive effect of miR-99a, we knockdowned mTOR in 786–0 cells and found that the proliferation and colony formation were decreased and the G1-phase population was increased, similar to the phenotype observed upon miR-99a restoration in 786–0 cells. However, the migration and invasion of mTOR-knockdowned 786–0 cells were not decreased, which suggests that the regulation of miR-99a on migration and invasion in RCC cells is not likely related to mTOR inhibition. There results suggest that the tumor suppressive role of miR-99a may be mediated partially through mTOR pathway regulation.

On the basis of these findings, we propose a hypothetical model for the function of the miR-99a–mTOR axis in RCC. Downregulation of miR-99a leading to increase of mTOR and p-mTOR results in the phosphorylation of 4E-BP1 and p70S6K, which in turn activates protein synthesis,promotes cell proliferation and cell clonability and allows progression from the G1 to the S phase of the cell cycle. It has been reported that miR-100 is downregulated and targets mTOR in clear cell ovarian cancer [29] and childhood adrenocortical tumors [20]. More recently, miR-199a-3p was also shown to be downregulated and target mTOR in hepatocarcinoma cells [30]. These characteristics of miR-100 and miR-199a-3p are quite similar to those of miR-99a, indicating that mTOR expression might be regulated redundantly by various closely related miRNAs. It is postulated that each miRNA regulates up to 100 different mRNAs and that more than 10,000 mRNAs appear to be directly regulated by miRNAs [31]. In our study, we found that the regulation of miR-99a on migration and invasion in RCC cells is not likely related to mTOR inhibition. Thus, it remains possible other targets might be at least partially involved. The mechanisms underlying miR-99a implicated in the carcinogenesis of RCC is very complicated, and further extensive analysis will be necessary to elucidate the precise mechanisms of miR-99a implicated in the carcinogenesis of RCC.

Expression of miR-99a has been proved frequently downregulated in various tumors [14-20], but the mechanisms underlying the downregulation of miR-99a in cancers remain to be unknown. It has been reported that downregulation of miR-99a is caused by the activation of Src/Ras-related pathways in human tumors [23]. The gene encoding miR-99a was found residing within an intron of C21or f34, C21 or f34 located in chromosome 21q21, the region was commonly deleted in lung cancer [13,32]. Recently, miR-99a was also shown to be co-transcripted with C21 or f34 in hepatocellular carcinoma [17]. Up to date, there are no studies on the mechanisms of miR-99a downregulation in RCC, so illuminating the mechanisms responsible for downregulation of miR-99a in RCC would be our next study in the future.

## Conclusions

In conclusion, our study demonstrates for the first time that deregulation of miR-99a is involved in the etiology of RCC partially via direct targeting mTOR pathway. In view of our present results showing decreased miR-99a expression in RCC clinical samples correlating with overall survival of RCC patients and the suppression of tumorigenicity upon upregulation of miR-99a in vitro and in vivo, we propose a hypothesis that miR-99a may be an attractive target for prognostic and therapeutic interventions in RCC.

## Competing interests

We declare that we have no conflict of interest.

## Authors’ contributions

LC, HZ and HZ carried out the experimental studies and performed the statistical analysis. LC drafted and completed the manuscript. YZ was in charge of the clinical samples selection and performed the proofreading. RX, XX, LZ, ZX, WX and BZ disposed the tissue samples. TD and YC completed sample conservation. ZT and QS refined the manuscript. XH conceived and designed of the study. All authors read and approved the final manuscript.

## Pre-publication history

The pre-publication history for this paper can be accessed here:

http://www.biomedcentral.com/1471-2407/12/546/prepub

## References

[B1] White NM Yousef GM MicroRNAs: exploring a new dimension in the pathogenesis of kidney cancer BMC Med 2010 8 65 10.1186/1741-7015-8-65 20964839 PMC2978114

[B2] van Spronsen DJ de Weijer KJ Mulders PF De Mulder PH Novel treatment strategies in clear-cell metastatic renal cell carcinoma Anticancer Drugs 2005 16 7 709 717 10.1097/01.cad.0000167901.58877.a3 16027518

[B3] Pascual D Borque A Epidemiology of kidney cancer Adv Urol 2008 2008 782381 19009036 10.1155/2008/782381 PMC2581742

[B4] Reeves DJ Liu CY Treatment of metastatic renal cell carcinoma Cancer Chemother Pharmacol 2009 64 1 11 25 10.1007/s00280-009-0983-z 19343348

[B5] Janzen NK Kim HL Figlin RA Belldegrun AS Surveillance after radical or partial nephrectomy for localized renal cell carcinoma and management of recurrent disease Urol Clin North Am 2003 30 4 843 852 10.1016/S0094-0143(03)00056-9 14680319

[B6] Chow TF Youssef YM Lianidou E Romaschin AD Honey RJ Stewart R Pace KT Yousef GM Differential expression profiling of microRNAs and their potential involvement in renal cell carcinoma pathogenesis Clin Biochem 2010 43 1–2 150 158 19646430 10.1016/j.clinbiochem.2009.07.020

[B7] Garzon R Pichiorri F Palumbo T Visentini M Aqeilan R Cimmino A Wang H Sun H Volinia S Alder H MicroRNA gene expression during retinoic acid-induced differentiation of human acute promyelocytic leukemia Oncogene 2007 26 28 4148 4157 10.1038/sj.onc.1210186 17260024

[B8] Schickel R Boyerinas B Park SM Peter ME MicroRNAs: key players in the immune system, differentiation, tumorigenesis and cell death Oncogene 2008 27 45 5959 5974 10.1038/onc.2008.274 18836476

[B9] Calin GA Croce CM MicroRNA signatures in human cancers Nat Rev Cancer 2006 6 11 857 866 10.1038/nrc1997 17060945

[B10] Huang Y Dai Y Yang J Chen T Yin Y Tang M Hu C Zhang L Microarray analysis of microRNA expression in renal clear cell carcinoma Eur J Surg Oncol 2009 35 10 1119 1123 10.1016/j.ejso.2009.04.010 19443172

[B11] Shenouda SK Alahari SK MicroRNA function in cancer: oncogene or a tumor suppressor? Cancer Metastasis Rev 2009 28 3–4 369 378 20012925 10.1007/s10555-009-9188-5

[B12] Iorio MV Croce CM MicroRNAs in cancer: small molecules with a huge impact J Clin Oncol 2009 27 34 5848 5856 10.1200/JCO.2009.24.0317 19884536 PMC2793003

[B13] Nagayama K Kohno T Sato M Arai Y Minna JD Yokota J Homozygous deletion scanning of the lung cancer genome at a 100-kb resolution Genes Chromosomes Cancer 2007 46 11 1000 1010 10.1002/gcc.20485 17674361

[B14] Nam EJ Yoon H Kim SW Kim H Kim YT Kim JH Kim JW Kim S MicroRNA expression profiles in serous ovarian carcinoma Clin Cancer Res 2008 14 9 2690 2695 10.1158/1078-0432.CCR-07-1731 18451233

[B15] Wong TS Liu XB Wong BY Ng RW Yuen AP Wei WI Mature miR-184 as potential oncogenic microRNA of squamous cell carcinoma of tongue Clin Cancer Res 2008 14 9 2588 2592 10.1158/1078-0432.CCR-07-0666 18451220

[B16] Gao W Shen H Liu L Xu J Xu J Shu Y MiR-21 overexpression in human primary squamous cell lung carcinoma is associated with poor patient prognosis J Cancer Res Clin Oncol 2011 137 4 557 566 10.1007/s00432-010-0918-4 20508945 PMC11828261

[B17] Li D Liu X Lin L Hou J Li N Wang C Wang P Zhang Q Zhang P Zhou W MicroRNA-99a inhibits hepatocellular carcinoma growth and correlates with prognosis of patients with hepatocellular carcinoma J Biol Chem 2011 286 42 36677 36685 10.1074/jbc.M111.270561 21878637 PMC3196113

[B18] Catto JW Miah S Owen HC Bryant H Myers K Dudziec E Larre S Milo M Rehman I Rosario DJ Distinct microRNA alterations characterize high- and low-grade bladder cancer Cancer Res 2009 69 21 8472 8481 10.1158/0008-5472.CAN-09-0744 19843843 PMC2871298

[B19] Sun D Lee YS Malhotra A Kim HK Matecic M Evans C Jensen RV Moskaluk CA Dutta A miR-99 family of microRNAs suppresses the expression of prostate-specific antigen and prostate cancer cell proliferation Cancer Res 2011 71 4 1313 1324 10.1158/0008-5472.CAN-10-1031 21212412 PMC3523179

[B20] Doghman M El WA Cardinaud B Thomas E Wang J Zhao W Peralta-Del VM Figueiredo BC Zambetti GP Lalli E Regulation of insulin-like growth factor-mammalian target of rapamycin signaling by microRNA in childhood adrenocortical tumors Cancer Res 2010 70 11 4666 4675 10.1158/0008-5472.CAN-09-3970 20484036 PMC2880211

[B21] Fingar DC Richardson CJ Tee AR Cheatham L Tsou C Blenis J mTOR controls cell cycle progression through its cell growth effectors S6K1 and 4E-BP1/eukaryotic translation initiation factor 4E Mol Cell Biol 2004 24 1 200 216 10.1128/MCB.24.1.200-216.2004 14673156 PMC303352

[B22] Grewe M Gansauge F Schmid RM Adler G Seufferlein T Regulation of cell growth and cyclin D1 expression by the constitutively active FRAP-p70s6K pathway in human pancreatic cancer cells Cancer Res 1999 59 15 3581 3587 10446965

[B23] Oneyama C Ikeda J Okuzaki D Suzuki K Kanou T Shintani Y Morii E Okumura M Aozasa K Okada M MicroRNA-mediated downregulation of mTOR/FGFR3 controls tumor growth induced by Src-related oncogenic pathways Oncogene 2011 30 32 3489 3501 10.1038/onc.2011.63 21383697

[B24] Petroulakis E Mamane Y Le Bacquer O Shahbazian D Sonenberg N mTOR signaling: implications for cancer and anticancer therapy Br J Cancer 2007 96 Suppl R11 R15 17393579

[B25] Guertin DA Sabatini DM Defining the role of mTOR in cancer Cancer Cell 2007 12 1 9 22 10.1016/j.ccr.2007.05.008 17613433

[B26] Vignot S Faivre S Aguirre D Raymond E mTOR-targeted therapy of cancer with rapamycin derivatives Ann Oncol 2005 16 4 525 537 10.1093/annonc/mdi113 15728109

[B27] Kapoor A Inhibition of mTOR in kidney cancer Curr Oncol 2009 16 Suppl 1 S33 S39 19478899 10.3747/co.v16i0.419 PMC2687803

[B28] Menon S Manning BD Common corruption of the mTOR signaling network in human tumors Oncogene 2008 27 Suppl 2 S43 S51 19956179 10.1038/onc.2009.352 PMC3752670

[B29] Nagaraja AK Creighton CJ Yu Z Zhu H Gunaratne PH Reid JG Olokpa E Itamochi H Ueno NT Hawkins SM A link between mir-100 and FRAP1/mTOR in clear cell ovarian cancer Mol Endocrinol 2010 24 2 447 463 10.1210/me.2009-0295 20081105 PMC2817607

[B30] Fornari F Milazzo M Chieco P Negrini M Calin GA Grazi GL Pollutri D Croce CM Bolondi L Gramantieri L MiR-199a-3p regulates mTOR and c-Met to influence the doxorubicin sensitivity of human hepatocarcinoma cells Cancer Res 2010 70 12 5184 5193 10.1158/0008-5472.CAN-10-0145 20501828

[B31] Hummel R Hussey DJ Haier J MicroRNAs: predictors and modifiers of chemo- and radiotherapy in different tumour types Eur J Cancer 2010 46 2 298 311 10.1016/j.ejca.2009.10.027 19948396

[B32] Yamada H Yanagisawa K Tokumaru S Taguchi A Nimura Y Osada H Nagino M Takahashi T Detailed characterization of a homozygously deleted region corresponding to a candidate tumor suppressor locus at 21q11-21 in human lung cancer Genes Chromosomes Cancer 2008 47 9 810 818 10.1002/gcc.20582 18523997

