# Correction to: MicroRNA-99a induces G1-phase cell cycle arrest and suppresses tumorigenicity in renal cell carcinoma

**DOI:** 10.1186/s12885-021-07839-z

**Published:** 2021-01-29

**Authors:** Li Cui, Hua Zhou, Hu Zhao, Yaojun Zhou, Renfang Xu, Xianlin Xu, Lu Zheng, Zhong Xue, Wei Xia, Bo Zhang, Tao Ding, Yunjie Cao, Zinong Tian, Qianqian Shi, Xiaozhou He

**Affiliations:** 1 Department of Urology, The Third Affiliated Hospital of Soochow University, 185 Juqian Street, Changzhou, 213003 China; 2 Department of Nephrology, The Third Affiliated Hospital of Soochow University, Changzhou, China; 3 Department of Urology, The Affiliated Jiangyin Hospital of Southeast University Medical College, Wuxi, China; 4 Comprehensive Laboratory, The Third Affiliated Hospital of Soochow University, Changzhou, China

**Correction to: BMC Cancer 12, 546 (2012)**

**https://doi.org/10.1186/1471-2407-12-546**

Following publication of the original article [1], the authors reported an error in Fig. 7e. The wrong images were placed in the original manuscript, when choosing representative images from the countless image data. This does not affect the figure legend, results and conclusions of the article. The corrected figure is presented in this correction article. The authors apologize for the error.

**Fig. 7:**
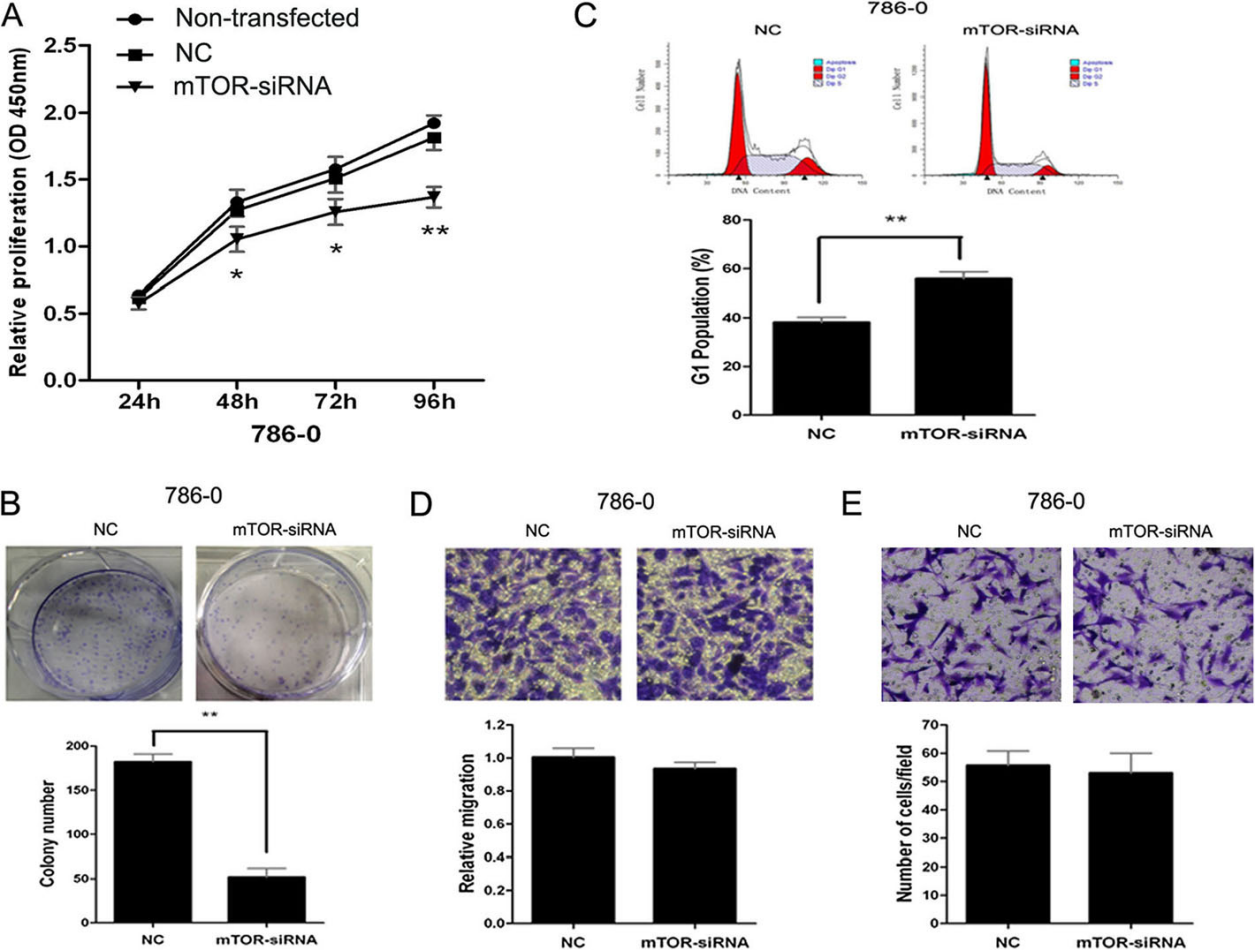
MTOR knockdown partially phenocopies miR-99a restoration in renal cell carcinoma cells. 786-O cells were transfected with mTOR-siRNA or NC followed by functional assays. Cell proliferation assay by CCK-8 (**a**), colony formation assay (**b**), cell cycle analysis by FACS (**c**), transwell-migration assay (**d**) and transwell-invasion assay (**e**) in 786-O cells transfected with mTOR- siRNA or NC. We also detected the proliferation of non-transfected 786-O cells. Data are represented as mean ± SD from three independent experiments. *, *P* < 0.05. **, *P* < 0.01
